# Asynchronous Teledermatology Reduces Wait Times and Maintains High Adherence to Standard Care for Lichen Planus: A Retrospective Study

**DOI:** 10.1177/26924366251361365

**Published:** 2025-07-24

**Authors:** Taylor L. Duffy, Joseph C. English

**Affiliations:** Department of Dermatology, University of Pittsburgh Medical Center, Pittsburgh, PA, USA.

**Keywords:** lichen planus, teledermatology, asynchronous, diagnostic concordance, retrospective

## Abstract

**Background:**

Lichen planus (LP) is a chronic inflammatory dermatosis with limited teledermatology data guiding its management.

**Objective:**

To assess whether asynchronous teledermatology provides timely, guideline-adherent LP care comparable to in-person visits.

**Methods:**

A retrospective study of 102 LP patients seen via asynchronous teledermatology (*n* = 45) or in-person (*n* = 57) from 2020 to 2024. Variables included demographics, response time, diagnostic concordance, treatment, and follow-up compliance.

**Results:**

Teledermatology provided rapid access (mean response time: 15 h) to dermatologic care. Diagnostic concordance between teledermatologists and in-person dermatologists was high (90.5%), while concordance with primary care providers was lower (18.5%, *p* < 0.001). Teledermatologists followed LP care standards, including hepatitis C virus screening (91.1%) and topical corticosteroid initiation (90%). Black patients used eVisits more frequently than in-person care (38.9% vs. 8.8%, *p* < 0.01), and follow-up compliance was lower among asynchronous patients (57.8% vs. 92.7%, *p* < 0.001).

**Conclusion:**

Asynchronous teledermatology enables timely, high-quality LP care. However, strategies to improve follow-up are needed, particularly for younger teledermatology patients.

## Introduction

Teledermatology has rapidly expanded in recent years, particularly asynchronous or “store-and-forward” models, which allow patients or referring providers to submit images and clinical histories for dermatologic review.^1^ This modality has been shown to improve health care efficiency by reducing wait times, minimizing resource misuse, and expanding access to specialty care, particularly in areas with a shortage of dermatologists.^1,2^ Notably, over two-thirds of U.S. counties had no practicing dermatologists as of 2013,^3^ with rural and nonmetropolitan areas experiencing the most pronounced shortages.^3,4^

A growing body of literature supports the reliability of teledermatology for both diagnosing and managing dermatologic disorders.^5,6^ Most studies report diagnostic accuracy rates for teledermatology in the 75–79% range, comparable to those achieved during face-to-face evaluations.^7–11^ In a systematic review of 20 studies involving a wide range of dermatologic conditions, diagnostic concordance between teledermatologists and in-person dermatologists ranged from 60% to 100%.^12^ Similarly, multiple studies have shown that treatment recommendations made via teledermatology match in-person evaluations in 70–90% of cases.^7,8,13–17^

Despite these successes, teledermatology studies focus narrowly on initial assessments, with limited data on follow-up outcomes or adherence to disease-specific management guidelines. The influence of patient demographics—such as age and race—on care delivery and outcomes also remains poorly characterized, limiting insight into potential disparities. Moreover, few studies directly compare asynchronous care models—namely, provider-to-provider models (eConsults) and patient-to-provider models (eVisits)—despite their distinct workflows and implications for clinical decision-making.

These gaps are consequential for chronic inflammatory dermatoses, which often require longitudinal care, ongoing monitoring, and strict adherence to treatment protocols to prevent morbidity and improve outcomes.^18,19^ While asynchronous teledermatology has proven to be effective in managing chronic inflammatory dermatoses such as psoriasis,^20,21^ atopic dermatitis,^22,23^ and rosacea,^24^ its role in lichen planus (LP)—a chronic inflammatory condition affecting ∼1% of the global population^25^—remains largely unstudied, representing a critical and timely gap in the literature.

LP can involve multiple mucocutaneous sites—including skin, oral mucosa, genitalia, nails, and scalp—and often requires longitudinal management with careful assessment of potential triggers, such as hepatitis C virus infection (HCV) and medication exposure.^25,26^ Diagnosing LP can be clinically challenging due to its morphological heterogeneity and overlap with other lichenoid dermatoses, making it an important condition to study in teledermatology settings.^25,26^

When left untreated, LP can result in significant morbidity. Oral and genital erosive variants can cause persistent pain, bleeding, and scarring, while scalp involvement may lead to irreversible hair loss.^25,26^ Of particular concern is oral LP, which carries an increased incidence of squamous cell carcinoma.^27,28^ These complications underscore the need for timely diagnosis, patient education, and longitudinal monitoring—needs that teledermatology may help meet, especially for patients with limited access to dermatologic care.

Given the gap in the literature regarding asynchronous teledermatology for LP and the success of this modality in managing other chronic inflammatory conditions, we conducted a retrospective analysis comparing asynchronous and in-person care for LP. We aimed to assess the impact of asynchronous teledermatology on access to care, workup, diagnosis, treatment, and follow-up compliance of patients diagnosed with LP compared to in-person patients with the same diagnosis.

## Methods

A retrospective analysis of electronic medical records from the University of Pittsburgh Medical Center between January 2020 and June 2024 identified 102 patients with LP. Fifty-seven cases were in-person visits, while 45 were asynchronous teledermatology visits divided into 27 physician-to-physician consults (eConsults) and 18 patient-to-physician visits (eVisits). For visual assessment, teledermatologists reviewed clinical photographs submitted by patients (eVisits) or consulting physicians (eConsults), while in-person dermatologists relied on real-time examinations. Data analysis, performed using StataSE 18.0 (StataCorp, College Station, TX), included demographics, consult response times, diagnostic concordance, workup, treatment, and follow-up compliance. For patients who were first evaluated via asynchronous teledermatology and later seen in person for the same condition, we compared the diagnoses from both visits to assess concordance between teledermatology and in-person evaluations. Similarly, for eConsults, diagnostic concordance was assessed between referring primary care providers (PCPs) and teledermatologists. Institutional Review Board (IRB) approval was granted by the University of Pittsburgh (STUDY22010151).

## Results

Descriptive statistics comparing in-person and asynchronous care are presented in Tables 1 and 2. Table 1 compares in-person visits to all asynchronous teledermatology visits combined, while Table 2 stratifies asynchronous visits into eConsults and eVisits to enable further analysis. The average consult submission to response time of 15 h (Table 1) contrasts with the several weeks patients typically wait for dermatological care,^29^ highlighting teledermatology’s ability to expedite access for LP patients. Of the 45 patients seen via asynchronous teledermatology, 21 (46.7%) were later evaluated in-person for the same condition. Among these, diagnostic concordance between the initial teledermatology assessment and subsequent in-person dermatology follow-up was 90.5% (Table 1), supporting the accuracy of asynchronous evaluation for LP. However, the low concordance (18.5%) between consulting PCPs and teledermatologists (Table 2) reveals a gap in diagnostic accuracy among PCPs for LP (*p* < 0.001), highlighting teledermatology’s role in bridging this gap. Among five eConsult patients with both cutaneous and oral involvement, concordance between teledermatology teams and PCPs was 60%, compared to 7% for those with solely cutaneous or oral involvement (*p* = 0.0128). This suggests that multiple sites of involvement enhance diagnostic concordance by providing additional clinical clues.

**Table 1.** Comparison of Demographic, Diagnostic, and Management Characteristics Between In-Person and Asynchronous Teledermatology Patients

Variable	In-person (*n* = 57)	Asynchronous (*n* = 45)	*p*
Age	Mean: 62.2	Mean: 52.9	<0.05
	Median: 64	Median: 55	<0.01
	Range: 20–90	Range: 10–85	N/A
	Standard deviation (SD): 16.1	SD: 16.6	Not significant (NS)
Gender (%)	Female: 68.4	Female: 57.8	NS
	Male: 31.6	Male: 42.2	
Race (%)			
White	80.7	62.2	NS
Black	8.8	22.2	<0.01
Asian	7	4.4	NS
Other/unspecified	1.8	2.2	NS
Ethnicity (%)			NS
Non-Hispanic	Non-Hispanic: 94.7	Non-Hispanic: 88.9	
Hispanic	Hispanic: 1.8	Hispanic: 0	
Unspecified	Unspecified: 3.5	Unspecified: 11.1	
Consult submission to response time (h)	N/A	Mean: 15	N/A
		Median: 11.6	
		Range: 0.2–84	
		SD: 16.7	
HCV screening (%)	86	91.1	NS
Patient compliance to HCV test (%)^a^	90.9 (*n* = 33)	81.5 (*n* = 27)	NS
Screening for drug-related cause (%)	100	97.8	NS
Sites of involvement (%)^b^	Cutaneous: 54.4	Cutaneous: 75.6	NS
	Oral: 39.3	Oral: 20	
	Genital: 14.3	Genital: 15.6	
	Nails: 12.5	Nails: 8.9	
	Scalp: 3.6	Scalp: 2.2	
Treatment modified (%)	86	95.5	NS
Topical corticosteroids as first-line treatment (%)	77.2	90	NS
Diagnostic concordance (teledermatology vs. in-person dermatology follow-up) (%)^c^	N/A	90.5 (*n* = 21)	N/A
Follow-up compliance (%)	92.7	57.8	<0.001

^a^ Subset of patients who were advised to undergo hepatitis C screening.

^b^ Values will not add up to 100% due to some patients having more than one site of involvement.

^c^ Subset of asynchronous patients with in-person dermatology follow-up.

HCV, hepatitis C virus; N/A, not applicable; NS, not significant; SD, standard deviation.

**Table 2.** Subgroup Comparison of eVisit and eConsult Patients Within the Asynchronous Teledermatology Cohort

Variable	Asynchronous-eVisit (*n* = 18)	Asynchronous-eConsult (*n* = 27)	*p*
Age	Mean: 45.1	Mean: 57.9	<0.05
	Median: 46	Median: 62	<0.01
	Range: 10–72	Range: 22–85	N/A
	Standard deviation (SD): 15.5	SD: 15.6	N/A
Gender (%)	Female: 61.1	Female: 55.6	Not significant (NS)
	Male: 38.9	Male: 44.4	
Race (%)			
White	50	70.4	NS
Black	38.9	3.7	<0.01
Asian	0	3.7	NS
Other/unspecified	11.2	11.1	NS
Ethnicity (%)			NS
Non-Hispanic	77.8	96.3	
Hispanic	0	0	
Unspecified	22.2	3.7	
Consult submission to response time (h)	Mean: 21.8	Mean: 10.4	<0.05 NS N/A
	Median: 3.9	Median: 18.46	
	SD: 10.5	SD: 21.7	
HCV screening (%)	88.9	92.6	NS
Patient compliance to HCV test (%)^a^	66.7 (*n* = 9)	88.9 (*n* = 18)	NS
Screening for drug-related cause (%)	100	96.3	NS
Sites of involvement (%)^b^	Cutaneous: 83.3	Cutaneous: 70.4	NS
	Oral: 11.1	Oral: 25.9	
	Genital: 16.7	Genital: 14.8	
	Nails: 5.6	Nails: 11.1	
	Scalp: 0	Scalp: 3.7	
Treatment modified (%)	94.4	96.3	NS
Topical corticosteroids as first-line treatment (%)	90	90	NS
Diagnostic concordance (consulting PCP vs. teledermatology) (%)^c^	N/A	18.52	<0.001
Diagnostic concordance (teledermatology vs. in-person dermatology follow-up) (%)^d^	88 (*n* = 8)	92.3 (*n* = 13)	NS
Follow-up compliance (%)	55.6	59.3	NS

^a^ Subset of patients who were advised to undergo hepatitis C screening.

^b^ Values will not add up to 100% due to some patients having more than one site of involvement.

^c^ Only applicable to eConsult patients.

^d^ Subset of asynchronous patients with in-person dermatology follow-up.

PCP, primary care provider.

Teledermatologists adhered to established standards of care for LP,^25^ screening for HCV infection (91.1%) and drug-related causes (97.8%), while prescribing topical corticosteroids as a first-line treatment in 90% of new cases (Table 1).

Demographic disparities were also observed, with a statistically significant difference between Black eVisit patients (38.9%, Table 2) and Black in-person patients (8.8%, Table 1) (*p* < 0.01). White patients comprised 50% of the eVisit group and 80.7% of the in-person group. Smaller percentages identified as Asian (0% eVisit vs. 7.0% in-person) or other/unspecified (11.2% eVisit vs. 1.8% in-person). While our data do not capture the reasons behind patients’ care modality selection, the higher proportion of Black patients in the eVisit group may suggest that asynchronous patient-to-physician platforms help reduce barriers to in-person care. Further research is needed to better understand these trends and to inform the design of care models that effectively support diverse patient populations.

As shown in Table 1, asynchronous teledermatology patients had a lower mean age than in-person patients (52.9 vs. 62.2 years, *p* < 0.05). Follow-up compliance was also significantly lower among asynchronous teledermatology patients compared to in-person patients (57.8% vs. 92.7%, *p* < 0. 001) (Table 1). A similar pattern emerged with HCV testing, where asynchronous teledermatology patients complied 81.5% of the time compared to 90.9% for in-person. Contributing factors may include challenges navigating asynchronous communication systems, difficulties accessing resources without in-person support, or reduced perceived need for follow-up.

## Conclusion

Our findings suggest that asynchronous teledermatology can provide timely, high-quality care for patients with LP, demonstrating comparable diagnostic accuracy and treatment adherence to traditional in-person dermatology. To the best of our knowledge, this is the first study to evaluate asynchronous teledermatology for LP—a chronic inflammatory dermatosis that affects ∼1% of the globe.^25^ Prior studies have established the efficacy of asynchronous care for other inflammatory conditions such as psoriasis, atopic dermatitis, and rosacea.^20–24^ Our results extend this evidence base by showing that asynchronous platforms also support guideline-concordant management of LP, including high rates of HCV screening (91.1%), drug-related etiology evaluation (97.8%), and corticosteroid initiation (90%) (Table 1).^25,26^

These findings align with prior teledermatology research emphasizing its capacity to reduce diagnostic delays, improve specialist access, and maintain therapeutic integrity, especially in underresourced settings.^1,2,4,6^ In our study, the average consult submission to response time via asynchronous teledermatology was just 15 h—markedly faster than the several weeks patients often wait for in-person dermatologic evaluation.^29^ This supports earlier findings that teledermatology can serve as a rapid triage tool to expedite care and reduce bottlenecks in dermatologic access.^24^

Diagnostic concordance between teledermatologists and in-person dermatologists (90.5%) reinforces the reliability of asynchronous assessments for LP, while the low agreement between PCPs and teledermatologists (18.5%) underscores the added diagnostic value of dermatology input in this condition. These findings are particularly noteworthy given LP’s diagnostic complexity and potential morbidity when inadequately managed.^25–28^ Asynchronous platforms may play a crucial role not only in access but also in improving diagnostic precision, especially when initial assessments are made by non-specialists in settings where dermatology access is limited.^3,4^

Our data also showed significantly lower follow-up compliance among teledermatology patients (57.8% vs. 92.7%). These patient engagement challenges have been discussed in previous chronic inflammatory teledermatology literature.^6,30^ This suggests that asynchronous platforms, while improving access, may face challenges with patient engagement and continuity of care, underscoring the need for tailored interventions—such as integrated care navigation—to ensure treatment completion and long-term follow-up.

The disproportionate use of asynchronous patient-to-physician (eVisit) care among Black patients also highlights the potential of teledermatology to reduce access barriers while also raising important questions about care-seeking behavior, trust in health care systems, and the types of support different patient populations may require.

Strengths of this study include its direct comparison between asynchronous and in-person care, its granular breakdown of asynchronous modalities (eVisits vs. eConsults), and its use of real-world clinical data from a large academic health system. Importantly, this is the first study to systematically evaluate asynchronous teledermatology for LP—a diagnostically challenging condition with high morbidity when inadequately treated. Additional strengths include the evaluation of multiple clinical and operational outcomes (e.g., diagnostic concordance, treatment adherence, follow-up compliance), as well as the identification of demographic trends in care modality usage, offering new insight into access disparities and practical considerations for implementation.

Several limitations must be noted. The retrospective design introduces potential biases in data capture and documentation. Additionally, the study was conducted within a single academic health system, which may limit generalizability to community-based settings or nonacademic teledermatology models. Having only 18 eVisits and 27 eConsults may also limit the robustness of subgroup analyses due to small sample size. Moreover, not all asynchronous patients received in-person follow-up, which restricts our ability to fully assess long-term outcomes and treatment efficacy. Finally, some in-person follow-ups were conducted by the same teledermatologists who rendered the initial asynchronous diagnoses, potentially introducing confirmation bias during in-person evaluation.

Future research should involve prospective multicenter studies to validate these findings, explore targeted interventions to improve follow-up adherence, and assess asynchronous teledermatology for other lichenoid dermatoses, such as lichen sclerosus. Incorporating patient-centered tools, such as automated reminders, digital education, or navigator support, may also enhance care continuity and optimize outcomes in asynchronous dermatologic care models.

## Abbreviations Used

eConsult: Asynchronous physician-to-physician electronic consultation
eVisit: Asynchronous patient-to-physician electronic visit
HPC: Hepatitis C virus
IRB: Institutional Review Board
LP: Lichen planus
N/A: Not applicable
NS: Not significant
PCP: Primary care provider
SD: Standard deviation
UPMC: University of Pittsburgh Medical Center
